# Disseminated *Mycobacterium abscessus* Infection Following Septic Arthritis: A Case Report and Review of the Literature

**DOI:** 10.1097/MD.0000000000000861

**Published:** 2015-05-29

**Authors:** Shoichi Fukui, Noritaka Sekiya, Yasunobu Takizawa, Hiroshi Morioka, Hirofumi Kato, Akio Aono, Kinuyo Chikamatsu, Satoshi Mitarai, Satomi Kobayashi, Satoshi Kamei, Keigo Setoguchi

**Affiliations:** From the Department of Rheumatology (SF, YT, S Kobayashi, S Kamei, KS); Clinical Laboratory (NS, HM, HK), Tokyo Metropolitan Cancer and Infectious Diseases Center, Komagome Hospital, Tokyo; and Department of Mycobacterium Reference and Research (AA, KC, SM), The Research Institute of Tuberculosis, Japan Anti-Tuberculosis Association, Kiyose, Japan.

## Abstract

*Mycobacterium abscessus* is a rapidly growing mycobacterium found mainly in patients with respiratory or cutaneous infections, but it rarely causes disseminated infections. Little is known about the clinical characteristics, treatment, and prognosis of disseminated *M abscessus* infection.

A 75-year-old Japanese woman who had been treated for 17 years with a corticosteroid for antisynthetase syndrome with antithreonyl-tRNA synthetase antibody developed swelling of her right elbow. X-ray of her right elbow joint showed osteolysis, and magnetic resonance imaging revealed fluid in her right elbow joint. *M abscessus* grew in joint fluid and blood cultures. She was diagnosed with a disseminated *M abscessus* infection following septic arthritis. Antimicrobial treatment by clarithromycin, amikacin, and imipenem/cilastatin combined with surgical debridement was administered. Although blood and joint fluid cultures became negative 1 week later, the patient died at 6 weeks from starting antimicrobial treatment.

We reviewed 34 cases of disseminated *M abscessus* infections from the literature. Most of the patients had immunosuppressive backgrounds such as transplantation, use of immunosuppressive agents, hematological malignancy, and end stage renal disease. The duration from onset of symptoms to diagnosis was over 3 months in half of the cases. All fatal cases had positive blood cultures or use of immunosuppressive agents.

Clinicians should bear in mind that mycobacterial infections including *M abscessus* are one of the differential diagnoses in patients with subacute arthritis and soft tissue infections.

## INTRODUCTION

*Mycobacterium abscessus* is a rapidly growing mycobacterium that exists ubiquitously in the environment, for example, in soil, dust, and water.^1^ A distinction between *M abscessus* and *M chelonae* became apparent only after 1992 with the advent of the polymerase chain reaction method.^2^ The clinical manifestations of *M abscessus*, which caused a gluteal abscess in a woman with osteoarthritis, were first described in 1953.^3^ The most common types of *M abscessus* infections are respiratory tract infections^4^ and localized skin and soft tissue infections.^5^ Disseminated *M abscessus* infection is a rare clinical presentation, and little is known about its clinical characteristics, treatment, and prognosis. Here we report a case of disseminated *M abscessus* infection following septic arthritis, and we provide a review of the literature.

## CASE PRESENTATION

A 75-year-old Japanese woman was admitted to our hospital complaining of swelling of the right elbow and the wrist joint for a month. She had no history of orthopedic surgery, joint trauma, or intraarticular injections in those joints. She had a 17-year history of dermatomyositis after being diagnosed at 58 years of age, along with interstitial lung disease (ILD), pulmonary hypertension, chronic kidney disease, and Raynaud phenomenon. She had pulmonary tuberculosis at the age of 33, resulting in old inflammatory changes and volume loss in the left lung. Later, her dermatomyositis proved to be antisynthetase syndrome with antithreonyl-tRNA synthetase antibody. Antisynthetase syndrome is characterized by the existence of antibodies to aminoacyl-transfer ribonucleic acid synthetase enzymes, myositis, ILD, arthropathy, fever, Raynaud phenomenon, and mechanic's hands.

The patient had remained on corticosteroids for 17 years; at least 3 times she had received high-dose corticosteroid therapy, that is, 45–60 mg prednisolone (PSL) per day continued for 4 weeks and tapered, with or without pulsed methylprednisolone (P-MPSL). As for other immunosuppressants, methotrexate was only temporarily used 10 years before this event; they were never used after that time. Four years before her present admission, she experienced the last exacerbation of antisynthetase syndrome-associated ILD that required the high-dose PSL therapy and P-MPSL. Despite the therapy, her respiratory function worsened due to the progression of fibrosis, and she had to receive home oxygen therapy. The PSL was tapered gradually to 10 mg per day over the next 3 years. She was on 9.5 mg PSL per day at the time of admission. On physical examination, she was afebrile, and all other vital signs were within normal limits. Her right elbow, forearm, and wrist joint were swollen with tenderness. Erythema and warmth were noted on her right arm. No skin eruptions or nodules were found. The cardiopulmonary and abdominal findings were normal.

**FIGURE 1:**
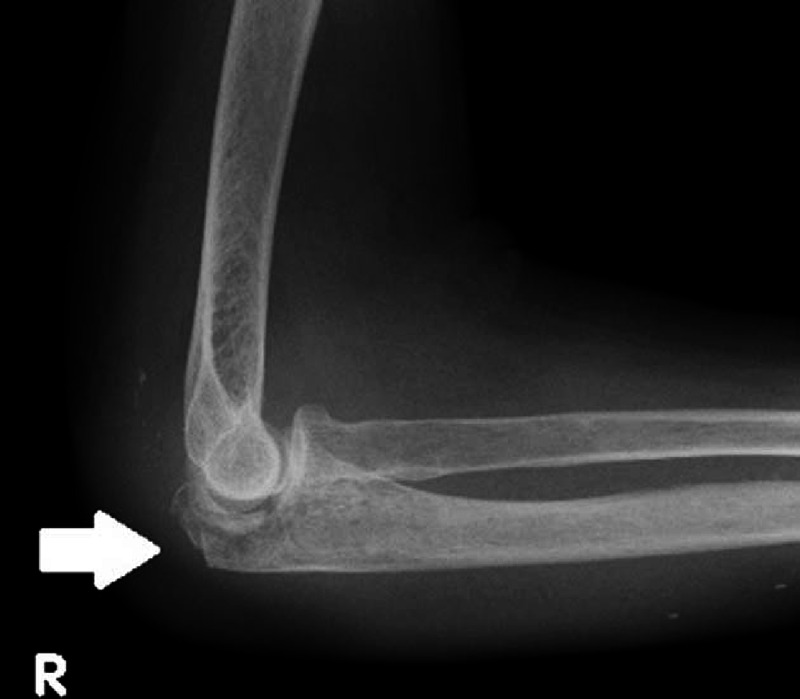
X-ray of the patient's right elbow joint. Osteolysis is seen (arrow).

**FIGURE 2:**
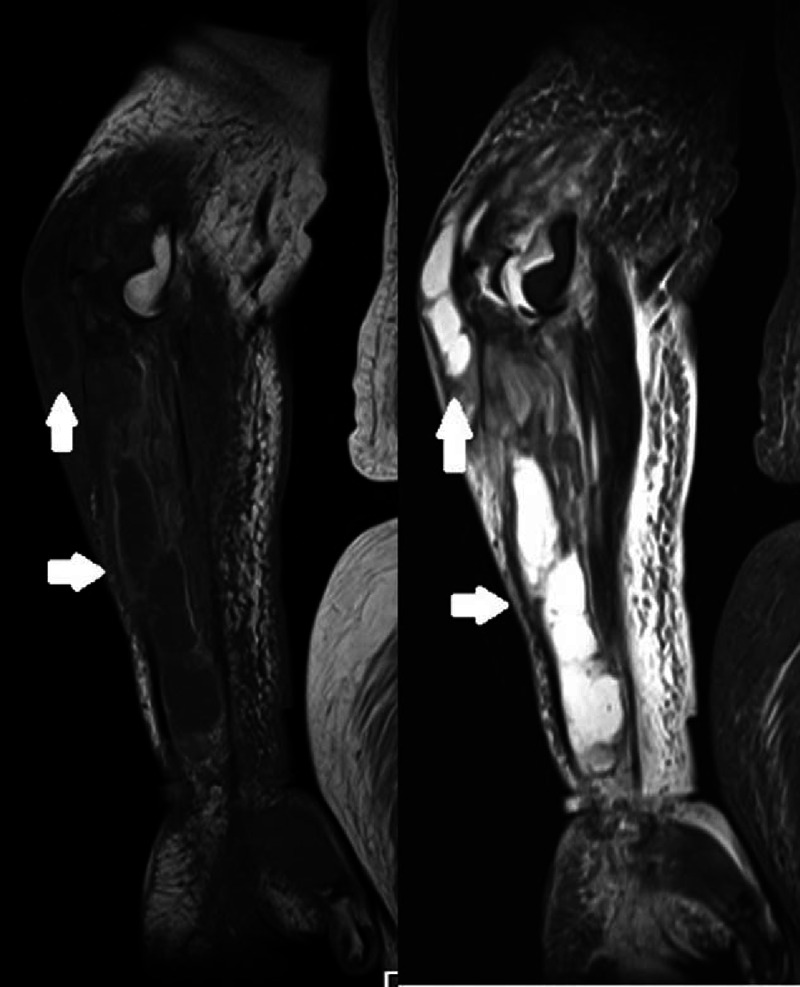
Magnetic resonance imaging shows fluid collection in the right elbow joint and forearm (arrows).

Laboratory examinations showed that white blood cell count was 9000/μL (normal range 3300–7600/μL, neutrophils 77%, lymphocytes 20%, monocytes 3%, and eosinophils 0%). Elevated serum levels of C-reactive protein (2.6 mg/dL, normal range <0.3 mg/dL) and creatinine (1.2 mg/dL, normal range 0.5–0.8 mg/dL, the estimated glomerular filtrating ratio was 34.0 mL/min) were shown. No other abnormalities were detected on laboratory testing. X-ray of her right elbow joint showed osteolysis and pathological fracture of the right olecranon (Figure 1). Magnetic resonance imaging revealed fluid in her right elbow joint and forearm (Figure 2). In addition, there were high-intensity lesions in the right olecranon, which suggested an osteomyelitis. *M abscessus*, identified by *hps65* gene sequencing (100% homology), grew in blood and joint fluid cultures (BacT/ALERT system; Biomérieux, Marcy-l’É toile, France).

Breakpoint susceptibility testing was performed using the broth microdilution method with 10 drugs based on the recommendations of the Clinical and Laboratory Standards Institute M24-A2^6^ (Table 1). Sputum cultures were negative for mycobacteria. A surgical debridement of the right elbow joint, forearm, and wrist joint was performed. The extra fluid in the patient's right elbow joint and forearm was completely removed. We initiated a vancomycin treatment for the patient, empirically. Based on the culture reporting, we switched the antimicrobial treatment to clarithromycin, amikacin, and imipenem/cilastatin. This combination therapy was continued for 6 weeks. The daily PSL was maintained to prevent adrenal insufficiency.

**TABLE 1:**
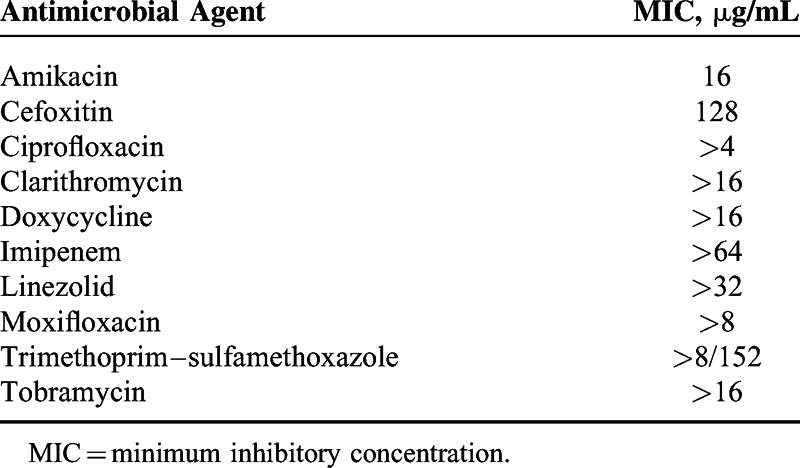
Minimum Inhibitory Concentrations Using the Broth Microdilution Method

The subsequent blood and joint fluid cultures became negative 1 week later. We placed a peripherally inserted central catheter in the right jugular vein for long-term intravenous antimicrobial therapy.

One month after the initiation of antimicrobials, the patient became febrile, and blood cultures turned positive for yeast-like fungi, which were identified as *Candida albicans*. The patient also had candida endophthalmitis. We added micafungin and later changed the micafungin to fluconazole based on the blood culture results. Although the subsequent blood cultures became negative for *C albicans*, a pleural effusion was increased with paroxysmal atrial fibrillation. The pleural fluid culture was negative. Two weeks later, the patient died due to respiratory failure possibly related to the disseminated candidiasis. An autopsy was not performed, per her family's request.

## DISCUSSION

Disseminated nontuberculosis mycobacterial infections in non-HIV-infected patients are considered uncommon.^7^ Although some cases were reported in the recent literature, there have been few large epidemiological or clinical studies of disseminated mycobacterial infections. Disseminated *M abscessus* infections such as that seen in our patient are extremely rare, and we therefore reviewed the past case reports and case series of disseminated *M abscessus* infections in non-HIV-infected patients by conducting a PubMed (http://www.ncbi.nlm.nih.gov/pubmed) search. Our search of reports from 1953 to 2014 used the search terms “dissemination,” “disseminated infection,” “*M abscessus*,” and “non-tuberculosis mycobacteria.”

Disseminated *M abscessus* infections are defined by at least one of the following characteristics: involvement of >1 organ, involvement of >2 groups of lymph nodes, or positive blood culture.^8^ We included cases that met these criteria. Table 2  shows the data obtained regarding the background, diagnostic process, and treatment for 34 patients.^8–27^

**TABLE 2:**
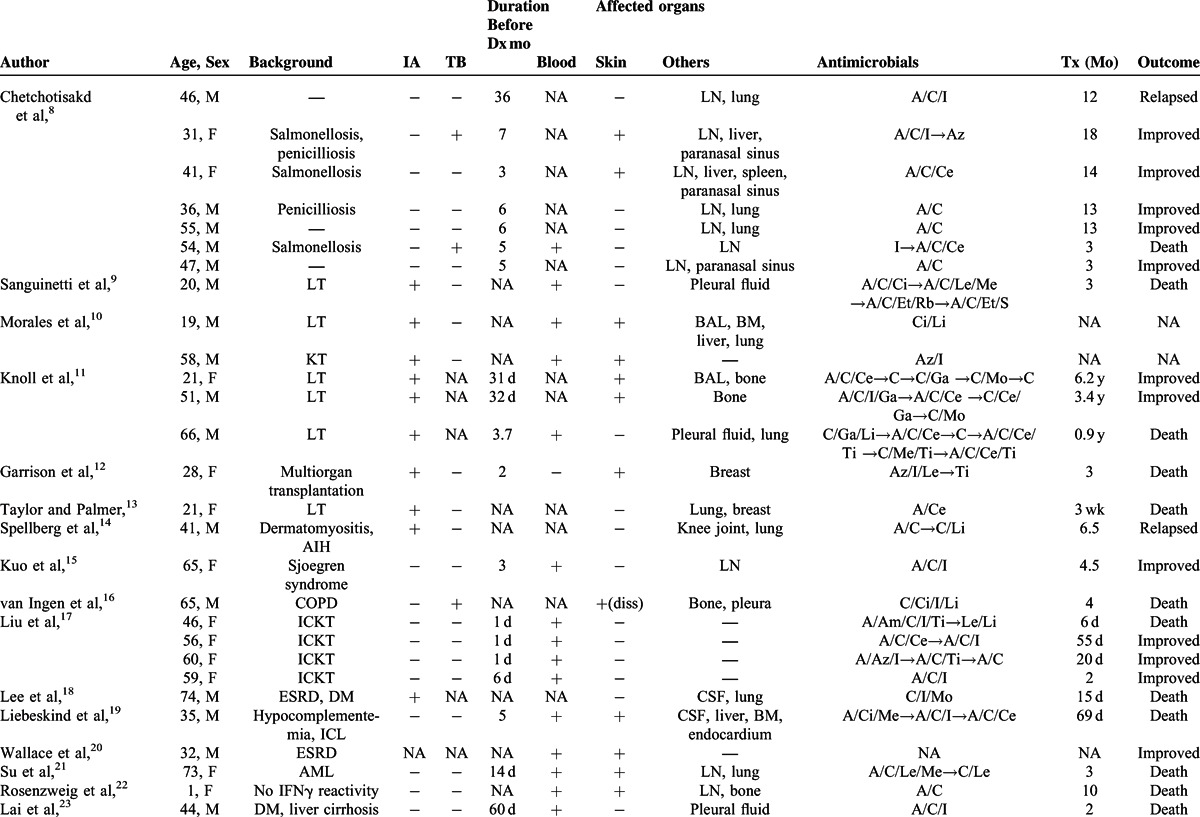
Demographic Data and Medical History of Disseminated *Mycobacterium abscessus* Infection Cases

**TABLE 2 (Continued):**
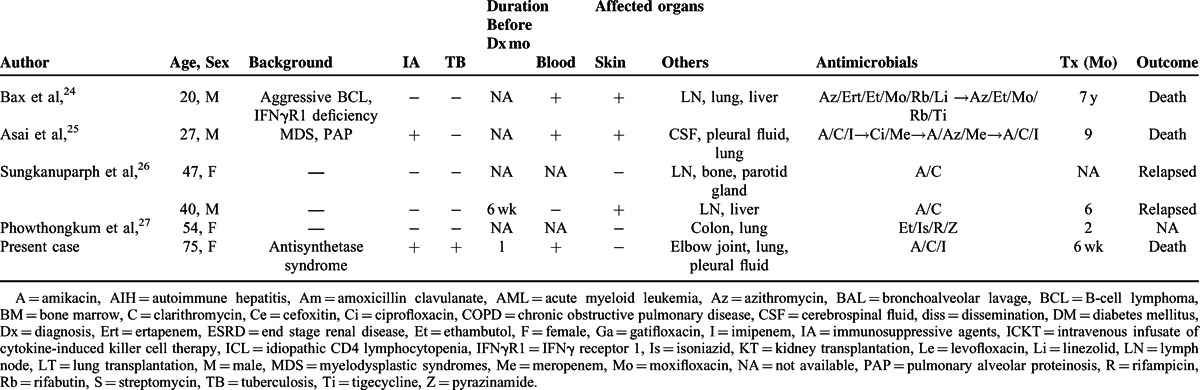
Demographic Data and Medical History of Disseminated *Mycobacterium abscessus* Infection Cases

Previous reports suggested that immunosuppressive backgrounds were risk factors, such as organ transplants^9–13^ and corticosteroid therapy for autoimmune diseases.^14,15^ Most of the patients listed in Table 2  had immunosuppressive backgrounds, 8 patients had a history of organ transplant, and 3 other patients received corticosteroid treatment. All these patients were in actively immunocompromised status, which meant the status with concurrent use of prednisolone or other immunosuppressive agents for visceral transplantations or autoimmune diseases at the time of infection. Our patient was treated with corticosteroid therapy for a long time, which could have been a risk for dissemination.

We also examined the patients’ history of tuberculosis.^8^ Four patients had a history of tuberculosis (Table 2 ). Specific cytokines, such as interleukin-12 and interferon gamma, are known to play an important role in the prevention of mycobacterial infections.^28^ However, it is not clear whether there was some potential vulnerability to mycobacterial infections in our patient.

She was diagnosed with disseminated infection 1 month after developing arthritis. Because mycobacterial infections typically show subacute disease progression, a delayed diagnosis may affect the progression to disseminated infections in some cases. Table 2  shows that the duration before diagnosis was >3 months in 48% of the cases (10/21, only assessed cases).

With regard to treatment, clarithromycin or azithromycin combined with parenteral medications (amikacin, cefoxitin, or imipenem) for serious infections is recommended.^29^ Of the parenteral antibiotics, amikacin is an important effective agent against *M abscessus*.^30^ Although clarithromycin is the cornerstone of therapy for *M abscessus*,^31^ clarithromycin-resistant *M abscessus* was reported, which was associated with *erm*(41) gene and *rrl* mutation.^32^ Because of the varying in vitro drug susceptibilities to some drugs, the antibiotic susceptibility testing of all clinically significant isolates is recommended.^29^ The prevalence of susceptibility of *M abscessus* to amikacin, clarithromycin, cefoxitin, and imipenem was reported to be 95%, 92.5%, 32.5%, and 12.5%, respectively.^33^

Although our patient was treated with clarithromycin, amikacin, and imipenem, her strain was susceptible only to amikacin and resistant to other antibiotics including clarithromycin and imipenem (Table 1). The subsequent cultures rapidly turned out to be negative, in contrast to the susceptibility. Multiple contributing factors could have led to respiratory failure and death, but the impact of *M abscessus* infection on the clinical course was unclear. Because a pleural fluid culture was negative for *M abscessus*, the pleural fluid may have been caused by either hypoalbuminemia caused by a continuous inflammation or heart failure. In addition, antisynthetase-syndrome-associated ILD or adverse effects of the antimicrobial combination therapy may have affected her respiratory failure.

The prognostic factors of disseminated *M abscessus* infections have not been well evaluated. As shown in Table 2 , 48% of the cases (15/31, only assessed cases) were fatal. Table 3 shows the clinical characteristics of the patients who died and those who survived. Although a statistical analysis was not conducted due to limitations of available data and selection bias, late diagnosis seems unrelated to poor prognosis.

**Table 3:**
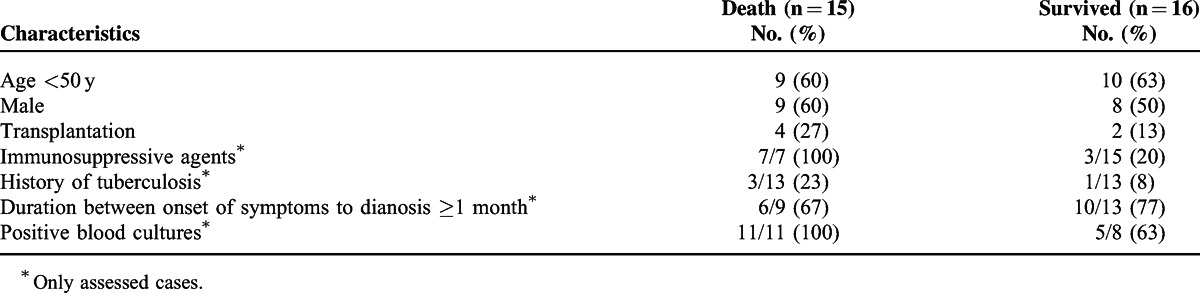
Comparison of Patients Who Died and Patients Who Survived

In contrast, cases with immunosuppressive agents and positive blood cultures are likely to be fatal. All fatal cases had received immunosuppressive agents or positive blood cultures (Table 3). These observations prompted us to hypothesize that bacteremia and immunosuppressive agents rather than late diagnosis might be associated with poor prognosis in disseminated *M abscessus* infections. The characteristics of our patient are consistent with this hypothesis; an immunocompromised host associated with prednisolone for antisynthetase syndrome with antithreonyl-tRNA synthetase antibody and positive blood cultures. Further evaluations are needed to elucidate the prognostic factors for individuals with an *M abscessus* infection.

## CONCLUSION

We described the case of a patient with a disseminated *M abscessus* infection following septic arthritis and provided a literature review. Disseminated *M abscessus* infections lead to poor prognosis, especially in patients with bacteremia and immunosuppressive agents rather than late diagnosis. Adequate clinical intervention to improve the outcome is unclear, but we need to be aware that mycobacterial infections including *M abscessus* should be included in clinicians’ differential diagnoses among patients with subacute arthritis and soft tissue infections.
